# Correlations between ERG, OCT, and Anatomical Findings in the *rd10* Mouse

**DOI:** 10.1155/2014/874751

**Published:** 2014-01-19

**Authors:** Sarah Rösch, Sandra Johnen, Frank Müller, Christiane Pfarrer, Peter Walter

**Affiliations:** ^1^Department of Ophthalmology, RWTH Aachen University, Pauwelsstraße 30, 52074 Aachen, Germany; ^2^Department of Anatomy, University of Veterinary Medicine Hannover, 30173 Hannover, Germany; ^3^Institute of Complex Systems, Cellular Biophysics, ICS-4, Forschungszentrum Jülich GmbH, 52428 Jülich, Germany

## Abstract

*Background*. To evaluate the correlation between ERG, OCT, and microscopic findings in the *rd10* mouse. *Methods*. C57BL/6J wild type mice and *rd10* mice were compared at the age of 2, 3, 5, 7, 9, 12, 24, and 48 weeks (each age group *n* = 3) using full-field electroretinography (ERG), spectral domain Optical Coherence Tomography (sd-OCT), fluorescein angiography (FA), Hematoxylin & Eosin histology (HE), and immunohistology (IH). *Results*. While in wild type mice, the amplitude of a- and b-wave increased with light intensity and with the age of the animals, the *rd10* mice showed extinction of the ERG beginning with the age of 5 weeks. In OCT recordings, the thickness of the retina decreased up to 9 weeks of age, mainly based on the degradation of the outer nuclear layer (ONL). Afterwards, the ONL was no longer visible in the OCT. HE staining and immunohistological findings confirmed the *in vivo* data. *Conclusion*. ERG and OCT are useful methods to evaluate the retinal function and structure *in vivo*. The retinal changes seen in the OCT closely match those observed in histological staining.

## 1. Introduction

Hereditary dystrophies of the retina, such as Retinitis pigmentosa (RP), are considerable causes of blindness in humans [1]. Research efforts concerning these currently not treatable diseases are focused on the genetic background, the mechanisms of degeneration, and possible treatment strategies [2]. Animal models in rodents, for example, the retinal degeneration *rd1* and *rd10* mice, are well characterized and described [3–9]. In *rd1* and *rd10* mice, missense point mutations in the gene encoding for the *β*-subunit of rod cGMP phosphodiesterase type 6 (*β*PDE) result in the described disease pattern [3–9]. The gene mutation leads to the degeneration of first rods and then consecutively cones with a central to peripheral gradient [9]. The fact that in both animal models firstly rods and secondly cones degenerate, which is comparable to RP [3–9], seems to be caused by missing diffusible factors normally secreted by the rods [10]. This kind of retinal degeneration is concomitant with a pronounced reduction in the thickness of the outer nuclear layer (ONL) [3–9]. The thickness of the inner retinal layers remains nearly unaffected [3–9]. The retinal development in the *rd1* and *rd10* mice is comparable to normal mice up to postnatal day 8 (P8) [2]. However, while in *rd1* mice, the degeneration becomes apparent at P11, in *rd10* mice, the degeneration starts at P16 and the peak of photoreceptor degeneration is reached at P25 [9]. By P60, no photoreceptors are left [9].

Because of the later onset and milder retinal degeneration, *rd10* mice seem to be more suitable to study slow disease mechanisms in RP [8]. Retinal vessels become sclerotic at 4 weeks of age and the a-wave and b-wave in the electroretinogram are visible with the age of 3 weeks and no longer detectable with the age of 2 months [8]. To characterize retinal degeneration, monitoring the time course of the disease is indispensable. Using histology to observe retinal changes requires a large number of animals to examine. Therefore, it is of interest, if data from functional examinations and *in vivo* imaging correlate well with histological data. We examined eyes of wild type and *rd10* mice in different age groups to determine retinal function by means of the full-field electroretinogram (ERG) as well as retinal thickness by means of spectral domain Optical Coherence Tomography (sd-OCT) scanning. The ERG examinations were performed under general anesthesia with ketamine and xylazine. This anesthesia was reported to have no influence on the oscillatory potentials and other ERG waveforms [11].

## 2. Materials and Methods

All experiments were performed in accordance with the ARVO Statement for the use of animals in ophthalmic and vision research and in accordance with the German Law for the Protection of Animals and after approval was obtained by the regulatory authorities. All possible steps were taken to avoid animal suffering at each stage of the experiment.

### 2.1. Animals

Adult pigmented wild type mice (C57BL/6J) and *rd10* mice were maintained under controlled light conditions (12 : 12 hours of light/dark cycle) with food and water available ad libitum. Animals (*n* = 3 in each group) were examined at the age of 2, 3, 5, 7, 9, 12, 24, and 48 weeks. At the end of the follow-up, animals at the same age were euthanized by isoflurane (Forene 100% (V/V), Abott GmBH, Wiesbaden, Germany) overdosing and decapitated for histological examinations.

For ERG, OCT, and fluorescein angiography, animals were anesthetized with an intraperitoneal injection of a mixture of ketamine (70 mg/kg Ketamin 10%, CEVA, Germany) and xylazine (10 mg/kg Xylazin 2% Bernburg, Medistar, Ascheberg, Germany) in 0.1 mL of saline.

### 2.2. Electroretinogram Recordings

Electroretinogram recordings (ERG) were performed with the Reti System designed for rodents (Roland Consult Electrophysiological Diagnostic Systems, Brandenburg, Germany). Mice were dark adapted for one hour and the pupils were dilated using 2.5% phenylephrine hydrochloride ophthalmic solution (Tropicamide 2.5% eye drops, Pharmacy of the University Hospital Aachen, Germany). After local anesthesia with proxymetacaine hydrochloride 0.5% eye drops (Proparakain-POS, Ursapharm, Saarbrücken, Germany), a custom-made goldring electrode (animal electrode 0.5 mm ø 3 mm Roland Consult) was placed on the corneal surface of each eye. Methylcellulose (Methocel 2%, Omni Vision, Puchheim, Germany) served for a good contact and to maintain corneal moisture. The reference goldring electrode was placed on the mouth mucosa. A subcutaneous silver needle electrode in the lumbar region served as ground electrode. The ERG was recorded as full-field ERG according to the standard protocol of the International Society for Clinical Electrophysiology of Vision (ISCEV) [12]. Five responses to light stimulation were averaged. a- and b-wave amplitude and implicit times of rod and cone responses were determined.

### 2.3. Spectral Domain Optical Coherence Tomography

Spectral domain Optical Coherence Tomography (sd-OCT) scans were performed using the Spectralis OCT system (Heidelberg Engineering, Heidelberg, Germany). To correct rodent optics, the system was modified according to recommendations of the manufacturer with a +25 D lens in front of the scanning system. Cross-sectional images centered on the optic disk as the main landmark were obtained from the retina. Retinal thickness was measured at six positions along the cross sectional image of the retina (maximum distance of 600 *μ*m from the optic nerve head) and averaged for each eye (Figure 2(b), red arrows). In the vast majority of cases, three positions on each side of the optic nerve head were chosen. However, as preliminary experiments showed, no thickness differences were observed, even if the six positions were taken on one side of the optic disk (Figure 2(b)). The thickness of the different retinal layers was determined using the same technique. Values represent mean ± SD. The confocal image (IR reflection image, wavelength 715 nm) of the fundus recorded by the OCT system was used for documentation.

### 2.4. Fluorescein Angiography

Fluorescein angiography was performed to evaluate the retinal vasculature. After dilatation of the pupils by Tropicamide 2.5% eye drops (Pharmacy of the University Hospital Aachen, Germany), images were taken by a confocal laser scanning microscope (Heidelberg Retina Angiograph-1, Heidelberg Engineering, Germany) in the fluorescein angiography mode. Shortly before the measurements, mice were intraperitoneally injected with 10–20 *μ*L fluorescein (Fluorescein Alcon 10%, Alcon Pharma GMBH, Freiburg im Breisgau, Germany).

### 2.5. Histology

Immunohistochemistry was performed as described earlier by Mataruga et al. [13]. In brief, eyes were enucleated and opened by an encircling cut at the limbus. The retinae in the eyecup were immersion fixed for 30 minutes in 4% paraformaldehyde (PA) in 0.1 M phosphate buffer (PB) at room temperature and washed in PB several times. Tissue was incubated in 10% sucrose in PB for 1 hour, followed by 30% sucrose over night. The retina was flat embedded and frozen in optimal cutting temperature compound (NEG-50, Richard Allen Scientific, Thermo Fisher Scientific, Germany). Vertical sections (i.e., perpendicular to the retinal layers; thickness of 20 *μ*m) were cut on a cryostat (HM 560 CryoStar, MICROM, Walldorf, Germany) and collected on Superfrost Plus slides (Menzel, Braunschweig, Germany). Sections were pretreated with blocking solution (5% chemiblocker (Chemicon, Hofheim, Germany), 0.5% Triton-X100 in PB, and 0.05% NaN_3_) for 1 hour, followed by incubation with primary antibodies over night, diluted in the same solution. Sections were washed in PB and incubated with secondary antibodies diluted in 5% Chemiblocker, 0.5% Triton-X100 in PB for 1 hour, washed in PB and coverslipped with Aqua Polymount (Polysciences, Eppelheim, Germany). Sections were examined with a confocal laser scanning microscope (TCS SP5 II; Leica Microsystems, Heidelberg, Germany) with 63x/1.4 oil immersion lenses. The following primary antibodies were used: against GFAP (anti-glial fibrillary acidic protein, raised in chicken, 1 : 2000; Novus, Germany); against CabP (anti-calbindin 28 K, raised in mouse, 1 : 1000; Sigma, Germany); against glutamine synthetase (raised in mouse, 1 : 4000; BD Biosciences, Germany); against PKC*α* (anti-protein kinase C*α*, raised in rabbit, 1 : 4000; Santa Cruz, USA); against calretinin (AB1550, raised in goat, 1 : 3000; Millipore, Germany); against HCN1 (RTQ-7C3, raised in rat, 1 : 10; F. Müller, Forschungszentrum Jülich, Germany); against rhodopsin (1D4, 1 : 500; R. S. Molday, Univ. of British Columbia, Canada); and against recoverin (AB5585, raised in rabbit, 1 : 2000; Millipore, Germany). Secondary antibodies included donkey anti-chicken Cy2 (1 : 400; Dianova, Germany), donkey anti-mouse Cy3 (1 : 100; Dianova, Germany), donkey anti-rabbit Cy2 (1 : 400; Dianova, Germany), donkey anti-rat Cy3 (1 : 500; Dianova, Germany), donkey anti-mouse Dy649 (1 : 500; Dianova, Germany), and donkey anti-goat Alexa647 (1 : 200; Invitrogen, Germany). Peanut agglutinin (PNA, biotinylated, 1 : 1600; Sigma Aldrich; Germany) was visualized using Streptavidin Alexa 647 (1 : 100; Invitrogen, Germany).

For Hematoxylin and Eosin (HE) staining, eyes were enucleated, punctured, and fixed by immersion as described above. Eyes were dehydrated in a tissue dehydration automat (mtm, Slee, Mainz, Germany), by incubation in a series of increasing ethanol concentrations (2x 70%, 2x 96%, and 3x 100% for 1 hour), followed by xylene (3x 1 hour) and paraffin (4x 1 hour), and embedded in paraffin. Sections of 5 *μ*m thickness were cut with a microtome (R. Jung, Heidelberg, Germany), collected on slides, deparaffinized, rehydrated, and stained with Hematoxylin and Eosin. Images were performed by a Leica DMRX microscope.

Because of the larger variability observed in retinal thickness measurements in HE-staining (probably due to the preparation process), we chose to determine retinal thickness in vertical sections stained for immunohistochemistry at six positions close to the papilla (comparable positions to the cross sectional images of the OCT, maximum distance of 600 *μ*m from the optic nerve head). For each retina, thickness values obtained from these positions were averaged. Values represent mean ± SD.

## 3. Results

**Figure 1:**
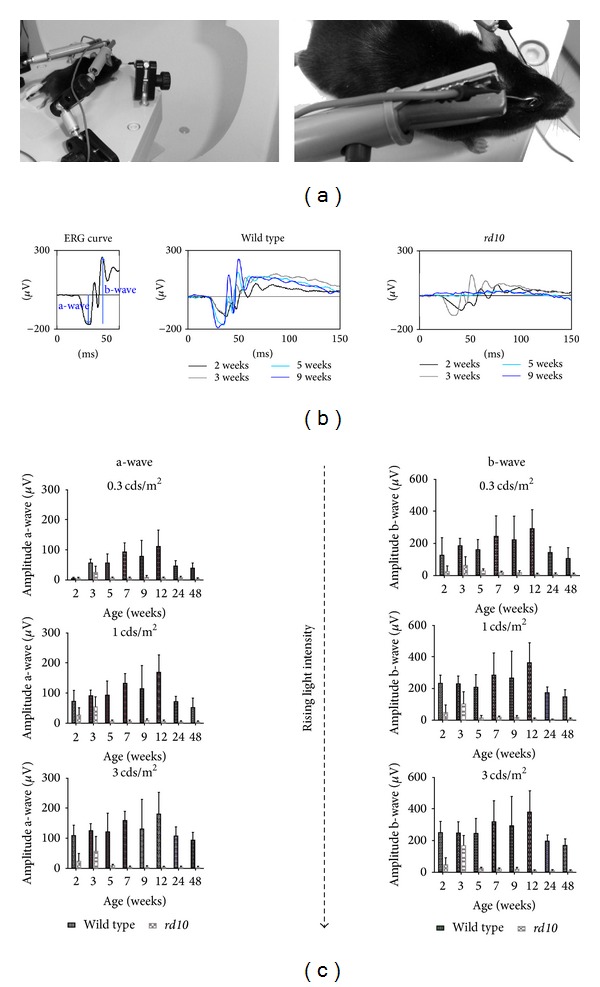
Electroretinogram recordings in wild type and *rd10* mice. (a) Left: mice were fixed in front of a Ganzfeld stimulator in a standardized position. Right: goldring electrodes were used on both eyes as active electrodes. A goldring electrode in the mouth served as reference and a subcutaneous needle electrode in the lumbar region as ground electrode. (b) The first graph serves as example for an ERG recording with designated a-wave and b-wave. In the following examples, ERGs of the left eye of wild type (second graph) and *rd10* mice (third graph) at different ages (2, 3, 5, and 9 weeks) are illustrated. While in wild type mice, the amplitudes of a- and b-waves increased with age up to 12 weeks, in *rd10* mice, an ERG was only recordable until the age of 3 weeks. (c) Amplitudes of the ERG's a-wave (left panel) and b-wave (right panel) in wild type mice (black bars) and *rd10* mice (grey bars) are plotted against the stimulus intensity (first row: 0.3 cds/m^2^, second row: 1 cds/m^2^ and third row: 3 cds/m^2^) and to age given on the abscissa. Vertical bars indicate the mean values ± standard deviation (*n* = 3 per age group).

ERG measurements were performed in wild type versus *rd10* mice, anesthetized with ketamine and xylazine using goldring electrodes as active electrodes instead of contact lenses (Figure 1(a)), in front of a Ganzfeld stimulator following a standardized protocol. The light-induced electrical activity of both eyes was recorded as full-field flash ERGs as described in the ISCEV standard protocol [12].

Typically, the ERG waveform (Figure 1(b)) consists of an early negative wave (a-wave; primary light response in photoreceptors), followed by large positive deflection (b-wave; dominated by the activity of ON-bipolar cells and Müller cells). Riding on top of the b-wave are the oscillatory potentials that probably involve inner retinal circuitry.

In wild type mice, the amplitude of a- and b-wave increased with light intensity and with age, reaching their maximal levels at the age of 12 weeks (Figures 1(b) and 1(c)). In *rd10* mice, amplitude increased only to the age of 3 weeks. In older *rd10* mice, the waves of the ERG were completely abolished (Figures 1(b) and 1(c)).

**Figure 2:**
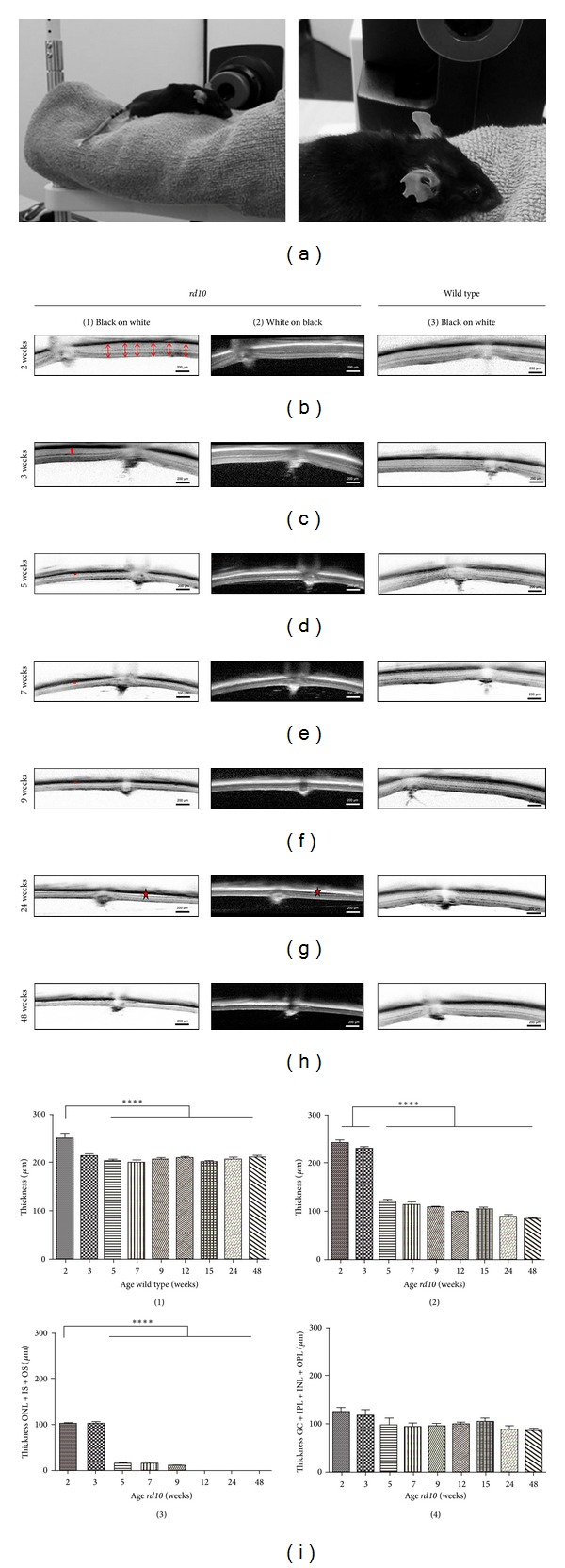
sd-OCT of wild type and *rd10* mice. (a) Set-up. The mouse is positioned in front of the magnifying lens of the OCT on the chin rest of equipment usually used for application in humans. ((b)–(h)) OCT images of wild type and *rd10* mice at different ages. All images are taken at the height of the papilla and displayed with the pigment epithelium to the top. Images of *rd10* mice are printed in black on a white background (first column) as well as in white on a black background (second column). Red arrows in (b) (first column) demonstrate the evaluation of thickness at the height of the papilla by six averaged cuts through the retina. Red bars in ((c)–(f)) (first column) mark the outer nuclear layer (ONL). In (g) (first and second column) red stars indicate a separation of the retina from the pigment epithelium. Separation was only observed *in vivo* in two animals of nine and 24 weeks of age, respectively (g). (i) Evaluation of retinal thickness in wild type and *rd10* mouse in the OCT. Values obtained from six cuts through the retina at the height of the papilla were averaged and plotted against the age (each age group: *n* = 3, both eyes per *n*). Thickness of the whole retina is illustrated in ((i)1) (= wild type) and ((i)2) (= *rd10* mouse). ((i)3): thickness of ONL (outer nuclear layer, somata) + inner and outer segments of the photoreceptors (IS + OS). In ((i)4), retinal thickness except photoreceptor layers is illustrated (GC = ganglion cell layer, IPL = inner plexiform layer, INL = inner nuclear layer, and OPL = outer plexiform layer). Values represent mean ± SD ((i)1) (wild type) for animals at the age of 2 weeks: ****P < 0.0001 versus animals older than 5 weeks; ((i)2) (*rd10*) for animals of 2 and 3 weeks ****P < 0.0001 versus animals older than 5 weeks; ((i)3) (*rd10*) for animals of 2 weeks ****P < 0.0001 versus animals older than 5 weeks; one-way ANOVA with Bonferroni's post hoc test).

Retinal thickness was measured and the fundus of the eye was observed *in vivo *by Optical Coherence Tomography (OCT) (Figure 2). For each retina, thickness was measured at six locations close to the optic disc (examples marked by red arrows in Figure 2(b), column 1). While in wild type mice, a decrease of thickness between the second and third week of age, followed by a relatively constant retinal thickness (Figure 2, column 3) was observed, in *rd10* mice, a significant loss of retinal thickness was obvious (Figure 2, columns 1 and 2). The outer nuclear layer (ONL, marked by red bars in Figures 2(c) and 2(f), column 1) was visible as a thin line up to 9 weeks. Retinal separation was only observed in one 9-week-old mouse—locally determined directly above the optic disc—and in one 24-week-old mouse near the papilla (Figure 2(g), first and second columns). In all of other animals of each age group (*n* = 3), no separation was found throughout the whole retina. In Figure 2(i), the averaged thickness values of the retina of wild type (Figure 2(i)1) and *rd10* mouse (Figure 2(i)2) determined in the OCT were plotted against the age in weeks. The loss of thickness in the *rd10* mice was mainly due to the thinning of the ONL (Figure 2(i)3), while the thickness of inner retinal layers stayed nearly constant (Figure 2(i)4). Up to the age of 9 weeks, the ONL thickness was reduced to 10.2% (thickness at 3 weeks: mean of 102.6 *μ*m; thickness at 9 weeks: mean of 10.44 *μ*m; *n* = 3 for each age group). All photoreceptor somata completely vanished at the age of 12 weeks.

**Figure 3:**
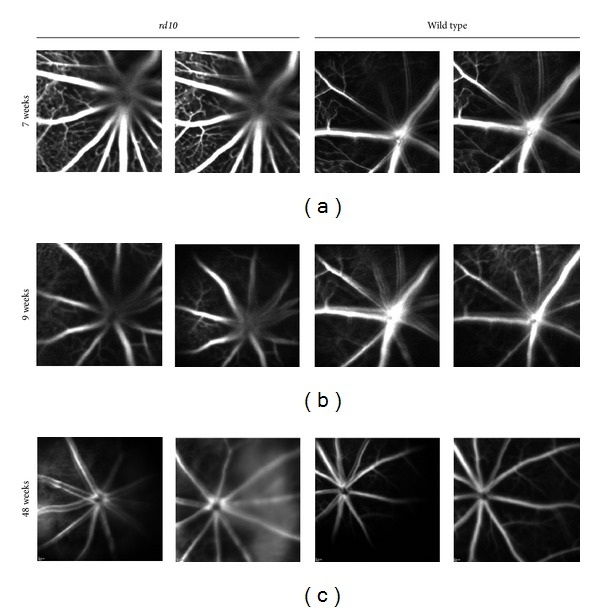
Fluorescein angiography in wild type versus *rd10* mice. In 7-, 9-, and 48-week old mice, no significant differences in vasculature could be observed between wild type and *rd10* after intraperitoneal application of fluorescein.

The retinae of the two strains were also examined using fluorescein angiography (Figure 3). No significant differences were observed.

**Figure 4:**
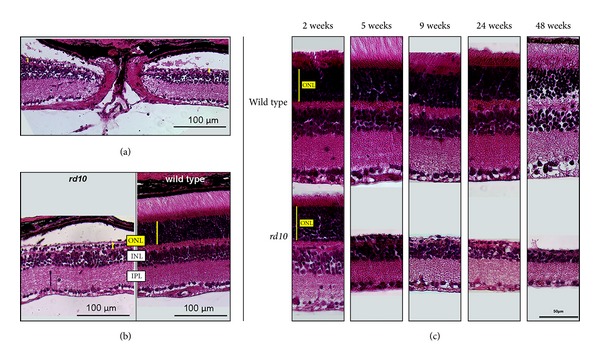
Histological evaluation of HE stained retinae. Pigment epithelium and photoreceptors are displayed to the top. (a) Area of evaluation. The papilla is cut directly through the median. (b) *rd10* mouse retina at the age of five weeks (left) in contrast to wild type mouse (right). In *rd10* mice, the ONL (yellow bars) was reduced to two layers of somata and the retina separated from the pigment epithelium. In wild type mice, outer segments of the photoreceptors were in contact with the pigment epithelium (same age; ONL = outer nuclear layer, INL = inner nuclear layer, and IPL = inner plexiform layer) (c) HE-staining of the retinae of wild type (first row) and *rd10* mice (second row) at the age of 2, 5, 9, 24, and 48 weeks. In *rd10* mice, the retinal thickness decreased with age, whereas in wild type mice, no differences in thickness were observed.

Results from the above reported non-invasive tests were compared to data obtained with histological techniques. For this evaluation, retinal areas close to the optic disc were chosen, comparable to the regions used for thickness measurements in the OCT (Figure 2). In contrast to wild type mice, in most of the *rd10* mice older than 3 weeks, artificial retinal separation was observed, probably based on the combination of photoreceptor degeneration and the preparation technique (Figure 4).

**Figure 5:**
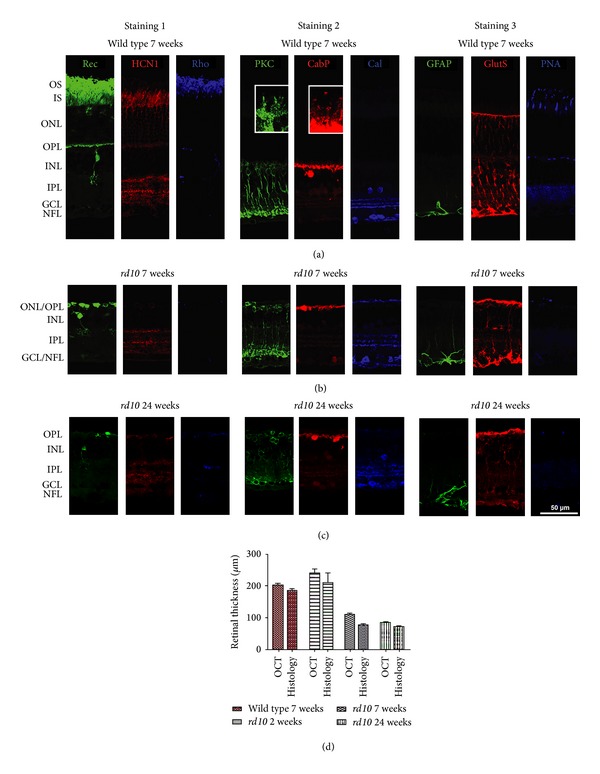
Immunohistochemical staining of wild type (a) and *rd10* retina at 7 weeks (b) and at 24 weeks (c). Staining 1: recoverin (all photoreceptors and type 2 bipolar cells) in green; HCN1 (photoreceptor somata and inner segments, processes in IPL, red); rhodopsin (rod outer segments, blue; puncta in the inner retina represent blood vessels labeled unspecifically by the secondary antibody). The ONL in the *rd10* mouse is considerably reduced to only one row of somata at 7 weeks (b) and completely lost at 24 weeks (c). Staining 2: rod bipolar cells (PKC*α*, green), horizontal cells (anti-28 kDa calcium-binding protein = CabP, red), and amacrine cells (calretinin, blue). Insets show processes of rod bipolar and horizontal cells at higher magnification. In *rd10* retina, such processes are lost. Staining 3: astrocytes (anti-GFAP, green), Müller cells (anti-glutamine synthetase = GlutS, red), and cone outer and inner segments (PNA, blue). In *rd10* mice, Müller cell processes are also GFAP-positive. Scale bar represents 50 *μ*m in overviews and 10 *μ*m in insets. Bar diagram in (d) illustrates retinal thickness evaluated *in vivo* by OCT (*n* = 3 per age group; both eyes per *n*) in comparison to measurements performed in immunohistochemistry (*n* > 6; both eyes per *n*). Thickness values *in vivo* and *in vitro* differ by 10 to 15%. Values represent mean ± SD.

In accordance with the results obtained with OCT, a decrease with age in the retinal thickness of *rd10* mice was observed in histology in HE staining (Figure 4) as well as in immunohistochemical stainings (Figure 5). In 5-, 7-, and 9-week-old *rd10* mice, the ONL was reduced to one row of photoreceptor somata. In the OCT measurements, thickness of wild type retinae (7 weeks) was determined as 203 ± 5.86 *μ*m, which agreed well with the thickness of 186.3 ± 11.26 *μ*m obtained from immunohistochemically stained sections at corresponding positions (Figure 5(d)). The histological work-up of the retinae may account for the observed differences in thickness of approximately 10 to 15% (Figure 5(d)).

To further investigate degenerative changes in the *rd10* mouse retina and to identify cellular changes, immunohistochemical techniques were employed using specific antibodies and confocal laser scanning microscopy (Figure 5). In Figure 5, different cell populations in the degenerated retina were visualized using three stainings with different combinations of antibodies. The results were consistent with those obtained by other methods: photoreceptor somata and outer segments degenerated (Figure 5, staining 1), while inner retinal cells such as bipolar cells, horizontal cells, and amacrine cells survived (Figure 5, staining 2). In 7-week-old wild type mice, 10 to 12 layers of somata were visible in the ONL (Figure 5(a)). In accordance with our findings in the HE staining, in *rd10* mice, the ONL was reduced to one row of somata at 7 weeks (Figure 5(b), staining 1, green) and completely vanished at 24 weeks (Figure 5(c), staining 1, green), while the thickness of inner retinal layers seemed to be unaffected (Figures 5(b) and 5(c)). In wild type retina, second-order neurons that contact rods like rod bipolar cells (Figure 5(a), staining 2, green) and horizontal cells (Figure 5(a), staining 2, red) display elaborate processes (see insets in Figure 5(a), staining 2). In *rd10* retinae, such processes are lost (Figures 5(b) and 5(c), staining 2). In wild type retina, GFAP expression was only found in astrocytes, while Müller cells were only positive for glutamine synthetase (Figure 5(a), staining 3). In *rd10* mice, Müller cells were also positive for GFAP (note the vertically oriented processes), indicating that they had become reactive during the retinal degeneration process (Figures 5(b) and 5(c), staining 3, green) [14].

## 4. Discussion

Animal models of retinal degenerations and dystrophies, such as the *rd10* mouse, are important to study the underlying disease mechanisms and also to establish possible treatment approaches [3–9]. No reduction of welfare of these animals over weeks was observed, although a loss of eyesight developed within the age of three weeks. Many studies on retinal degeneration rely on the combination of data obtained from large populations of experimental animals, sacrificed at different stages of the degeneration process. Recently, non-invasive tools such as ERG, sd-OCT, and fluorescein angiography have become promising methods that might allow following retinal degeneration in individual animals. In the present study, we show that the combination of these methods allows to precisely describe the degeneration of the retina *in vivo* during the course of the degenerative process.

The data we present here were obtained by non-invasive methods and are in accordance with findings of other groups [8, 11]. The values of a- and b-wave in the ERG were comparable to the results of other research groups [8, 9, 11]. Angiographic findings in the vasculature revealed no significant differences between wild type and *rd10* mice.

Most importantly, our OCT scans proved useful for quantitative analysis. The thickness of the ONL or of the entire retina measured *in vivo* by OCT scans was very well comparable to the thickness values obtained from histology.

Pennesi et al. [15] found a separation between the outer retina and the pigment epithelium (neurosensory detachment) in the retina of *rd10* mice at the age of 64 days. In our series of OCT scans, we were not able to confirm this finding. *In vivo*, we found neurosensory detachment only locally in two animals. However, after OCT, when the eyes were prepared for histology, separation was commonly observed probably induced during the histological work-up. The difference between our results and those of Pennesi et al. [15] might be explained by differences in the type of OCT devices. Alternatively, our results may indicate that, although separation is not always manifest *in vivo*, it can be more easily induced in the *rd10* mouse eye than in wild type eyes.

We employed two histological methods that complemented one another. For HE-staining, the whole eye was sectioned, which enabled us to also evaluate other parts than the neural retina. As a disadvantage, sectioning the entire eye is more prone to artifacts such as retinal separation that we observed especially in older *rd10* mice. Immunohistochemistry provides the advantage that the major neuronal and glial cell classes of the retina can be visualized in much detail using cell type specific antibodies. Our immunohistochemical results confirmed the early degeneration of rod and cone photoreceptors, the reactivity of Müller cells, and neuronal remodeling processes in certain types of bipolar and horizontal cells [16].

## 5. Conclusions

In summary, our data indicate that noninvasive techniques such as sd-OCT scan of the retina in rodents are powerful tools to monitor the course of retinal degeneration with respect to morphometric analyses and at the same time reduce the number of animals sacrificed for histological techniques. Quantitative anatomic parameters can be extracted from sd-OCT scans and are in good accordance with morphometric data from histological work-up. However, single cells and their involvement in the degenerative mechanisms can only be identified by immunohistochemical techniques optimally combined with confocal fluorescence imaging technology.
